# Profiles of Burnout, Job Demands and Personal Resources among Emergency Call-Takers and Dispatchers

**DOI:** 10.3390/healthcare10020281

**Published:** 2022-01-31

**Authors:** Maciej Załuski, Marta Makara-Studzińska

**Affiliations:** Division of Health Psychology, Faculty of Health Sciences, Collegium Medicum Jagiellonian University; Kraków 31-008, Poland; marta.makara-studzinska@uj.edu.pl

**Keywords:** emergency call-taker and dispatcher, perceived stress, occupational burnout, self-efficacy, person-oriented approach

## Abstract

According to scientific research, emergency call-takers and dispatchers are particularly vulnerable to burnout syndrome. There are no data describing specific burnout patterns or allowing for the definition of subgroups of workers who are particularly at risk. The aim of this research was to apply a person-oriented approach to characterize burnout profiles using job-related variables and personal resources. A cross-sectional survey study was conducted on 553 call-takers and dispatchers aged between 19 and 65, from 14 public safety answering points in Poland. The Link Burnout Questionnaire, the 10-item Perceived Stress Scale, the Generalized Self-Efficacy Scale, and an independent questionnaire were used to gather information. K-means cluster analysis was used, which allowed us to highlight three distinct burnout risk profiles: high risk of burnout, without full-blown pattern of burnout with high inefficacy, and no risk of burnout with an increased sense of disappointment. Several variables which coexisted with occupational burnout included work experience, weekly working hours, intensity of perceived stress, and self-efficacy level. The application of a person-oriented approach made it possible to identify groups of call takers characterized by a high risk of burnout syndrome, and to indicate the areas in which preventive measures, focused on each of their specific needs, should be taken.

## 1. Introduction

Occupational burnout is a multidimensional construct connected with the employee’s experience in the context of their professional work. According to the definition of the World Health Organization [1], burnout is a syndrome conceptualized as resulting from chronic workplace stress that has not been successfully managed. It is characterized by three dimensions: feelings of energy depletion or exhaustion; increased mental distance from one’s job or feelings of negativism or cynicism related to one’s job; and reduced professional efficacy [1].

Studies into burnout syndrome tend to apply one of two methodological approaches. The first one focuses on an analysis of relationships between groups of respondents with extreme scores obtained in particular dimensions of burnout (with the application of cut-off scores) and variables considered to be predictors. It is a variable-oriented approach. The subject of the analysis includes inter-individual variation and linear relationships between variables [2]. A disadvantage of this research method is the fact that it focuses only on data from groups which confirm the presence of burnout symptoms in each of its dimensions, ignoring the importance of the cases which do not follow the full-blown pattern. Additionally, focusing on respondents who obtained the results that allow for diagnosing burnout excludes, from further analysis, people who scored medium and high in only certain dimensions of burnout. However, this disadvantage can easily be overcome with an approach that focuses on analyzing the results for each dimension of burnout separately, and presenting the overall data in the form of burnout profiles (person-oriented approach). This second method allows for recognizing and comparing hidden burnout profiles in the given professional group. Detecting and analyzing hidden profiles allows for the verification of assumptions about the chronology of the incidence of burnout symptoms and the trajectory of their changes [3,4], in addition to identification of burnout profiles inconsistent with one pattern.

According to Maslach, most studies have tended to reduce burnout syndrome to a homogenous pattern and have not discriminated between subgroups according to their scores obtained in different burnout dimensions [4]. Burnout syndrome is believed to not have common symptoms which occur in all respondents, but is characterized by a lack of consistent etiology, and that the population of employees is heterogeneous in terms of the average level of burnout changes and one full-blown pattern of burnout [5,6]. According to Berjot et al., the diagnosis of burnout and the assessment of its prevalence should not be made solely with the use of cut-off scores [5]. The person-oriented approach to burnout syndrome makes it possible to observe groups which are inaccessible by the classic approach to burnout categorization (low, medium, high) [4], and recognize individualized ways of experiencing the conditions of the work environment. Due to this method, relationships between burnout and various working environment conditions were identified and could explain the cause and result relationships between burnout dimensions, and, considering the chronology of changes, made it possible to better plan preventive measures. This second method, initiated by research on a group of nurses, is also used in other professional groups [4,5].

One of the models used for explaining the reasons of professional burnout is the Job Demands–Resources Model of Burnout [7]. According to scientific research, requirements imposed on employees at work and the organizational resources helping to achieve professional goals, reducing requirements and connecting them with physiological and psychological costs constitute two separate clusters of work environment features, with various relation to occupational burnout and commitment to work. Organizational resources including providing employees with feedback on the results of their work, creating opportunities to improve professional skills, social support from the team after stressful phone calls, autonomy to make decisions, and creating opportunities for professional development, can act as a buffer against the relationship between work demands and burnout, and are predictors of withdrawal from interpersonal relations in the workplace [8]. Research has shown that an employee’s personal resources also belong to the organizational resources, which help to achieve professional goals and buffer the influence of requirements on an employee’s exhaustion. Personal resources are also believed to be potential predictors of the development of organizational resources [7,9]. The influence of work demands might be, to some extent, determined by one’s personal resource, as it is a perception of work stressors, in categories of their predictability, control, and sources of overload [10]. Perceived stress is an outcome variable measuring the experienced level of stress as a function of appraisal of objectively stressful events, coping processes, and personality factors [11] (p 386). Psychological stress is influenced by both the objective features of a situation (work requires constant cognitive, emotional, or physical effort) and dispositional variables, including a subjective evaluation of effort undertaken during struggles with work stressors, daily adversities and sense of self-efficacy. Self-efficacy, another personal resource, is defined as one’s belief in the possibility of achieving an intended goal, a belief that one is able to carry out a certain activity and achieve a successful result [12,13,14]. A high level of self-efficacy allows an ability to cope in a difficult situation in work, and positively cope with stressors [7]. It is a personal resource supporting demanding actions that require persistence and are characterized by a high level of complexity [15]. Researchers noticed that personal resources partially mediated the relationship between work resources and commitment to work, protecting workers from burnout. Regarding the three burnout components (emotional exhaustion, depersonalization and personal accomplishment), the strongest negative relationship between self-efficacy and burnout dimensions was found for lack of accomplishment [16]. Therefore, it is believed that positive self-efficacy can alleviate the negative effect of work environment demands and increase the desired impact they may have on employees’ commitment [7,17].

Emergency call-takers and dispatchers (ECDs) are positioned as working in more peripheral jobs compared with other emergency service workers. It is reflected in both the limited amount of scientific research devoted to this population and in the narrow scope of knowledge about this profession. At the same time, this profession is considered to be particularly stressful [18,19,20]. The main indicated sources of stress include being a witness to other people’s suffering, especially as they expect immediate help, the necessity to make responsible decisions in dynamically changing situations, and maintaining emotional neutrality in addition to reacting fast, without the possibility of checking the effects of the actions taken [21,22]. Other predictors of burnout connected with the job requirements of ECDs include: shift work leading to the lack of physical activity, poor nutrition and obesity [22], insufficient number of breaks for regeneration, exposure to traumatizing phone calls, exposure to verbal aggression of callers and colleagues, work which provokes strong emotional overload performed under time pressure with only short breaks between stressful phone calls, lack of sufficient support after stressful phone calls, and an insufficient number of staff training sessions devoted to preventive mental health protection [23]. Factors pertaining to the organization of working hours and shifts of work affected burnout in such professional groups, including firefighters, physicians, nurses and ECDs [23,24,25,26,27]. Rotating shifts and their accompanying inadequate sleep, and long hours of being sedentary, can be associated with psychological distress [23,27]. It has been well-documented that people who sleep less than 6 h per day are at higher risk of developing burnout syndrome [28]. Shift work may generate problems in maintaining a balance between work and family commitments, while conversely, it may offer the opportunity for ECDs to gain solitude during nonwork hours, which may allow ECDs to relax after work time [29]. Research findings suggest that age is negatively associated with burnout, or the relationship is bi-modal: burnout is elevated in both younger and older workers [30]. According to age and seniority, some studies have confirmed that the highest risk of burnout occurs at the beginning of a worker’s career, and decreases with age and seniority [5,25]. Santinello [31] assumed that people with less seniority are particularly exposed to burnout—due to the painful realization that their idealistic expectations prove very different from reality. Gender moderates the age–burnout relationship. Women are more exposed to work stressors and higher work–family conflicts [29]. In a study of hospital personnel, five work-related burnout profiles were revealed. The high-risk group was characterized by a younger age, greater female representation, with less seniority within the hospital, than the low risk group. The emotionally exhausted group was characterized by older age, more seniority and more satisfaction at work [32]. An additional source of stress in ECDs’ work might also be a lack of management support, and feedback and decision-making autonomy [23]. As a result of these factors, a decline in recruitment and job retention has been observed in recent years, and candidates have resigned from the job, even before the end of the training session, due to early symptoms of burnout [33].

ECDs are a particularly understudied emergency service population. There are not enough studies providing information which would help to match health prevention actions to the requirements of this professional group. The person-oriented approach presents opportunities, but no studies in the literature have utilized this approach to exploring burnout for a group of ECDs. While designing the study, we assumed that the application of a person-oriented approach would provide important information to complement the data provided by a variable-oriented approach. It is likely that there will appear groups of people who do not show full symptoms of burnout. Moreover, there does not exist any sufficient study on employees’ personal cognitive resources, such as the way of assessing a situation in terms of perceived stress, the level of self-efficacy, and the level of knowledge defined by respondents’ education. In order to overcome these deficiencies, this study was conducted with a focus on two objectives:

1. Differentiation of burnout profiles in the ECDs group, using four dimensions of burnout from the LBQ questionnaire, and describing the profiles of workers who are at risk of occupational burnout;
2. Determining the relationships connecting given profiles with some jobs demands, such as the number of shifts per month and working hours per week, and personal resources, such as the way of assessing the situation, the level of self-efficacy, the level of education and having an active hobby.

The following research hypotheses were formulated:

1. The application of a person-oriented approach provides information allowing for the determination of the risk of occupational burnout in ECDs;
2. Burnout profiles can highlight the differences in job demands and employees’ personal resources, which coexist with symptoms of burnout.

Our article presents the results of a study that was part of a larger research plan concerning a group of Polish ECDs. The entire research plan was used to verify many research hypotheses, the results of which have been presented in other articles [34,35].

## 2. Materials and Methods

### 2.1. Participants

The results presented in the article were drawn from a survey conducted between January and May 2020, on a group of ECDs working at 14 public safety points (PSAP) in Poland. Anonymous questionnaire sets were sent to 800 ECDs working in all 17 PSAPs. Completed sets were received from 558 respondents from 14 PSAPs. A total of 553 were completed correctly, which was 66.2% of all sent sets of questionnaires. The studies described in this article were part of a larger research plan and included a group of 543 ECDs (43.6% males and 56.4% females). In terms of age and other demographic variables, the group was characterized as having a: mean age of 34.43 years (SD = 8.11, range of years between 19 and 65); marital status—43.3% married, 26.0% cohabitated with a partner, 21.5% single, 8.3% divorced, 0.5% widowed, 0.4% missing data; and level of education—73.3% bachelor’s and master’s degree, 25.8% secondary education, 0.2% vocational education, 0.7% missing data. In terms of duration of service: 0–2 years for 26.9% of participants, 3–4 years for 24.0%, 5–7 years of service for 36.4%, over 7 years of service for 11.7% of participants, and 1% missing data. In terms of the number of shifts per month in the study group: up to 13 shifts declared for 11.6% of participants, 14–15 shifts for 69.2% of participants, and over 15 shifts for 19.2% of participants.

### 2.2. Instruments

The level of occupational burnout was assessed by the Link Burnout Questionnaire (LBQ) [31]. This questionnaire evaluates the severity of burnout symptoms according to 4 dimensions named: psychophysical exhaustion (PE), relationship deterioration (RD), professional inefficacy (PI), and disappointment (DI). We used versions of LBQ adopted for the Polish population by the Laboratory of Psychological Tests of the Polish Psychological Society. The instrument consists of 24 items with 6 Likert-type response options (1—never, 2—rarely, 3—once (or more) during a month, 4—more or less once a week, 5—several times a week, 6—every day). The PE subscale evaluates an employee’s psychophysical resources. The extreme end of the dimension expresses a state of exhaustion, fatigue and a feeling of being under pressure, and the opposite end, a state of activity and vital energy. The RD dimension evaluates the quality of interpersonal relations with service recipients (people seeking help). The extreme end of the dimension expresses the treatment of service recipients with objectivity, indifference or even hostility towards them, and the opposite end expresses commitment to relationships and individual treatment of each caller. The PI dimension is related to the evaluation of professional competences of the worker. The extreme end of the dimension expresses a sense of ineffectiveness and lack of work results, the opposite end expresses effectiveness in the work performed and efficiency in the pursued professional goals. The DI dimension evaluates the employee’s existential expectations towards the work performed. There are people who treat helping as a mission to help others. However, contact with professional reality can be disappointing. The extreme end of the dimension describes disappointment and lack of enthusiasm, while the opposite end describes passion, enthusiasm and job satisfaction. The LBQ includes 5 indicators: the higher the score of each subscale, the greater the intensity of each of the 4 dimensions of burnout, being between 6 points (low severity of burnout) and 36 points (maximum severity). The 5th indicator of burnout is occupational burnout syndrome composite index (LBQ^INDEX^), being the total burnout result on all dimensions (min—24 points, max—144 points). The Polish version of the LBQ questionnaire had adequate psychometric properties, Cronbach’s α ranged from PI = 0.628; RD = 0.689; to PE = 0.845; DI = 0.859. The LBQ questionnaire was used instead of the Maslach Burnout Inventory because it examines the additional (4th) dimension of burnout—disappointment.

The Perceived Stress Scale (PSS-10) [36] was used in the study. The PSS-10 questionnaire is a measure of assessing of perception of the cognitive aspects of stress and coping—appraising the effectiveness of coping strategies. The PSS-10 measures the unpredictability of live situations, lack of control over what is happening, and the feeling of being overloaded by circumstances [11]. The PSS-10 asks ten questions about feelings and thoughts over the past month, to which the respondent answers on a 5-point Likert-type scale (0—never, 1—almost never, 2—sometimes, 3—quite often, 4—very often). The overall raw result ranges between 0 and 40 points, with the higher the score, the greater the intensity of perceived stress. A score over 20 points indicates high perceived stress [11]. The Polish version of the PSS-10 questionnaire had adequate psychometric properties, with higher values in reliability, Cronbach’s α = 0.883.

The Polish version of the Generalized Self-Efficacy Scale (GSES) was used to measure beliefs about self-efficacy [37]. The questionnaire evaluates the strength of an individual’s general belief in their effectiveness in coping with difficult life situations and smaller, daily hassles. The instrument consists of 10 questions with 4 Likert type response options (1—not, 2—probably not, 3—probably yes, 4—yes). The overall raw result scores were between 10 and 40 points; the higher the score is, the greater intensity of the generalized self-efficacy it indicates. The Polish version of the GSES questionnaire had adequate psychometric properties, with higher values in reliability, Cronbach’s α = 0.883.

A uniform time frame, the past month, was established for the respondents to evaluate their experience in each questionnaire.

### 2.3. Other Factors and Data Analysis

We controlled the following demographic variables in the study: age, marital status, education, and active hobbies. The quantitative data were analyzed using software environment for statistical computing, R (version 4.1.1. The R Foundation for Statistical Computing, Vienna, Austria) [38]. Statistical significance was set at 0.05. Between-group differences were analyzed using the chi-square test (with Yates’ correction for 2 × 2 tables), and analyses of differences between three or more groups were performed using the Kruskal–Wallis H test. Once statistically significant differences were detected, the Dunn–Bonferroni post-hoc test for multiple analyses (15 variables) was performed.

## 3. Results

### 3.1. Group Characteristics according to Applied Variables

Table 1 presents the group characteristics according to the variables applied in the study. The respondents were between 26.3 and 42.5 years old. With regard to work experience in an ECD position, the group consisted of respondents who had worked from 6 months to 14 years, with the majority of people with working up to 5 years (66.3%), compared with those with working 6–10 years (31.5%), and more than 10 years (2.2%). The respondents worked from 3 to 28 shifts a month, being, on average, 14 shifts (42.9% of respondents) and working 40 h per week (27.7% of respondents). The group was dominated by people living in domestic partnership (70% of respondents) with higher education (70%). A total of 68.05% of respondents were trained in a job not related to the medical sciences, whereas 21.4% had only worked in an ECD job. Among the medical workers (10.55% of all respondents), 4.3% were paramedics by profession. A total of 91.1% of respondents declared that they had an active hobby. The results obtained from the examined group in the 4 dimensions of burnout were on the average level of: PE— 6 stanines, RD—7 stanines, PI—7 stanines, and DI—6 stanine. The mean value of the perceived stress measurement ranged between 3 sten scores (low) and 8 sten scores (high). The mean value of self-efficacy assessment ranged between 5 sten scores (average) and 9 sten scores (high).

### 3.2. Profiles of Burnout

**Figure 1:**
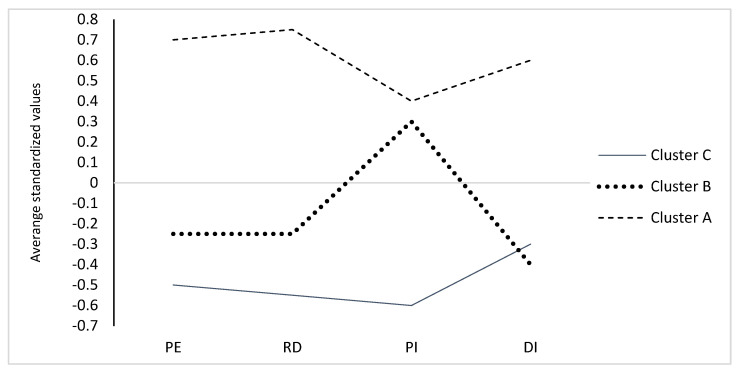
Characteristics of the 3 clusters solution. (*n* = 553). Note: standardized values were: 0—the value is equal to the mean in the studied group, 1—the value higher than the mean by one standard deviation, 1—the value lower than the mean by one standard deviation; variables were expressed as: PSS-10—perceived stress scale, GSES—generalized self-efficacy scale, PE—psychophysical exhaustion, RD—relation deterioration, PI—professional inefficacy, DI—disappointment.

Before analysis, the data were standardized and the convergence of their distribution with normal distribution was checked. As the LCA (Latent Class Analysis) method, used in the case of a person-oriented approach, was created with a view toward grouping observations described by qualitative variables (analysis of frequency tables and cross tables) [39], and in our study the data were mostly numerical, the clustering method was used for grouping the cases described in a quantitative way. In order to choose the best method of searching for clusters, three methods were taken into consideration: agglomerating hierarchical clustering with complete linkage [40], k-means clustering method [41], and partitioning around medoids [42]. It was determined that the number of clusters should range from two to six, as a larger number might be difficult to interpret. Seven measures of goodness of fit were chosen. Subsequently, a ranking of three methods was performed, taking into account seven measures of goodness of fit [43]. The k-means method and the squared Euclidean distance turned out to be the best in this comparison. Although it is recommended to perform one analysis by entering all predictors together, we decided to use only dimensions of burnout from the LBQ questionnaire as variables for the cluster analysis, similar to other studies [5]. The observation was divided onto three clusters: A (*n* = 189 cases), B (*n* = 182 cases) and C (*n* = 182 cases). Euclidean distances between clusters were: A—B: 2.16; A—C: 2.4, and B—C: 1.94. Figure 1 and Table 2 present characteristics of the three class solution. Respondents assigned to particular clusters differed in terms of the four dimensions of burnout: PE: χ^2^ = 132.57, *p* < 0.015; RD: χ^2^ = 123.96, *p* < 0.015; PI: χ^2^ = 72.76, *p* < 0.015; DE: χ^2^ = 87.27, *p* < 0.015, in addition to LBQ^INDEX^: χ^2^(2) = 292.093, *p* < 0.015. Cluster A (*n* = 189; 34.17% of the sample) consisted of respondents who showed particularly high rates of occupational burnout in each of the four dimensions, hence it was called the high risk of burnout group. Cluster B (*n* = 182; 32.1% of the sample) consisted of people whose occupational burnout indicators were below the mean for the whole examined group, apart from very high scores in the PI dimension. This cluster was called the group without full-blown pattern of burnout with high inefficacy. Cluster C (*n* = 182; 32.91% of the sample) consisted of respondents whose occupational burnout indicators were low. They were characterized by the lowest index of the loss of occupational efficacy and a relatively increased level of disappointment, hence it was called the group with no risk of burnout with an increased sense of disappointment.

### 3.3. Diversification of Profiles due to Sociodemographic Variables, Working Conditions and Personal Resources

Table 3 presents a comparison of the results of respondents belonging to a particular group, taking into account explanatory variables. There was a significant difference between the groups of respondents in terms of the length of working in an ECD position (χ^2^ = 43.25, *p* < 0.015). People from the group without full-blown pattern of burnout with high inefficacy had worked as an ECD significantly longer than people from the high burnout risk group (Cohen’s d = 1.01) and the group named as no risk of burnout with an increased sense of disappointment (Cohen’s d = 0.58). People from the burnout risk group definitely worked the shortest period of service compared with the other employees (Cohen’s d A- B = 0.97; Cohen’s d A-C = 0.41). There was no difference between clusters in terms of respondents’ average age, however, there was a statistically significant difference between them in the respondents’ sex (*p* < 0.015). The percentage of women was the highest in the without full-blown pattern of burnout with high inefficacy group of respondents, and slightly lower in the burnout risk group. Men definitely predominated in the no risk of burnout with an increased sense of disappointment group. There was no difference between the groups concerning the number of shifts per month, and their average number was 14, whereas the number of working hours per week was statistically significantly different between the groups (*p* < 0.015). The biggest number of working hours per week was observed in the case of respondents from the without full-blown pattern of burnout with high inefficacy group (Cohen’s d B − A = 0.46; Cohen’s d B − C = 0.42), whereas the respondents from the other two groups worked significantly less, and they did not differ between each other in this respect. Respondents’ level of education did not differ between particular groups (χ^2^ = 3.21, *p* < 0.75). The number of people with secondary education was the biggest in the burnout risk group and the lowest in the no risk of burnout with an increased sense of disappointment group. In people with higher education this relationship was the opposite, as the biggest number of people with this type of education was observed in the no risk of burnout with an increased sense of disappointment group, and the lowest number in the burnout risk group. The groups did not differ in terms of the profession they learned. With regard to personal resources, the results of measuring perceived stress were significantly different between particular groups (χ^2^ = 205.70, *p* < 0.015). Respondents from the without full-blown pattern of burnout with high inefficacy group assessed their level of perceived stress as 7 sten scores (high result), respondents from high burnout risk group as 5 sten scores (average result), whereas respondents from the no risk of burnout with an increased sense of disappointment group assessed their level of perceived stress as 4 sten scores (low result). The level of self-efficacy (χ^2^ = 59,31) and having an active hobby (χ^2^ = 9.08) differentiated the groups in a statistically significant way (*p* < 0.015). In the case of both of these variables their highest intensity was identified in the respondents from the no risk of burnout with an increased sense of disappointment group, then in those from burnout risk group and finally in respondents from the without full-blown pattern of burnout with high inefficacy group (Cohen’s d C − A = 0.40; Cohen’s d A − B = −0.56). The average score for self-efficacy in the case of respondents from the no risk of burnout with an increased sense of disappointment group reached 8 sten scores, in the group with burnout risk, 7 sten scores, and in the without full-blown pattern of burnout with high inefficacy group, 6 sten scores. There was no difference between respondents as far as their marital status was concerned.

## 4. Discussion

### 4.1. Introduction

The aim of the study was to identify occupational burnout profiles in ECDs and to differentiate them according to selected job requirements and personal resources. It was also important to identify people with the highest risk of occupational burnout. Before discussing the result, it is worth taking a look at the entire group of the participants of the study. A characteristic feature is a slight predominance of women, which seems to be significant, taking into account the demands of this job position. It is a common phenomenon in many countries around the world that women predominate among the employees of ECD centers. Sometimes it is even more common than in our study [22,27,44]. When it comes to other demographic variables, on the basis of the scarce scientific publications devoted to this problem, it may be concluded that the work of call-takers and dispatchers is usually performed by people aged between 25 and 45 with secondary or higher education [27,44]. Similar conclusions were reached in our study.

### 4.2. Burnout, Perceived Stress and Self-Efficacy among Emergency Call-Takers and Dispatchers

The scores obtained while assessing occupational burnout reached the upper limit of the average results, or even exceeded this limit. Following the guidelines of the LBQ author, we can conclude that these facts indicate the possibility of the incidence of some issues related to occupational burnout in addition to a high level of burnout in selected dimensions. Similar results were obtained in the studies conducted in other countries [22,44], which confirms a high risk of occupational burnout in the discussed profession.

The examined group was characterized by an average level of perceived stress, with results ranging between the low level and the border between the average and high level. The risk of a high level of stress in Polish ECDs is also observed in studies carried out on French call-takers and dispatchers, many of whom also complained of the symptoms of post-traumatic stress disorder [44], and in American operators for whom incidents of aggression from callers were quite common [45]. Results from other studies show the correlation of the level of perceived stress with the respondent’s sex and age [25,46,47]. There are few studies that have explored the relationship between the perceived stress, which precedes the physiological response, and other psychological processes. However, some research has shown that perceived stress can lower the efficiency of some cognitive functions [47].

The examined ECD group was characterized by scores of self-efficacy assessment reaching the border between the average and high levels. In this case, no comparable studies conducted in the discussed occupational group were found. However, in comparison with employees working in other professions involving high emotional burden, the average score was lower, for example over three points lower than the results obtained in the studies conducted on Polish air traffic controllers [48], almost three points lower than in the case of Polish maritime navigators and nearly two points lower than among Polish professional firefighters [25]. It is worth mentioning, however, that the three aforementioned occupational groups were dominated by men.

### 4.3. Profiles of Burnout among Emergency Call-Takers and Dispatchers

Moving on to discussing the essential results of the study, it is worth stating that the cluster analysis method resulted in distinguishing three different profiles of call-takers and dispatchers in accordance with the initial hypothesis. The first group of operators was characterized by strong burnout rate in all four dimensions of the construct. They were people on the verge of emotional exhaustion with a cynical or even hostile attitude towards the callers, with a clear sense of professional inefficacy, and bitterly disappointed with their work. According to JD-R theory, it can be explained by too high job demands [7].

The second group consisted of people with a low rate of emotional exhaustion, cynicism and disappointment, but with a clearly marked decrease in professional efficacy. These respondents reported a lack of efficacy and result of the work performed. They assessed their professional competences as very low, declared their insufficient self-efficacy, and an inability to face professional problems, even experiencing a sense of professional failure [31]. According to JD-R theory, it can be explained by too few job resources and personal resources [7]. Such a situation may be interpreted as both a manifestation of burnout, which in this case is not clearly visible in the other dimensions, and also as an employee’s inability to focus on minor changes, which could protect him or her from a decrease in their self-efficacy, lower commitment to work [31] and lack of sufficient job resources [8].

The third group included respondents with no symptoms of emotional burnout or lack of commitment, who still maintained a sense of professional efficacy. A characteristic feature of this group was a relatively increased level of disappointment with their work, slightly higher than in the second group, but definitely lower than in the group with high risk of burnout.

### 4.4. Profiles of Burnout, Organizational Demands and Personal Factors

Moving on to the discussion relating to the second hypothesis, we will focus on the intergroup differences arising from job demands. Work demands such as time pressure, emotional stress caused by an employee’s interactions with clients, overload, work–home conflict or physical work demands are predictors of the incidence of health problems and emotional exhaustion in employees [49]. Considering two specific demands examined in the study, the number of shifts per month and number of working hours per week, only in the second case were the initial assumptions confirmed. Although an increase of shifts per month was expected to result in a higher risk of burnout, especially emotional exhaustion, this assumption was not confirmed. It was probably due to the fact that the number of shifts per month is similar in various ECD units, hence there are no differences between separate groups. Conversely, what turned out to be essential was the way of organizing work in terms of number of hours per week. It was observed that a burden in the form of a high number of working hours per week may coincide with a decrease in self-efficacy, as it was observed in the without full-blown pattern of burnout with high inefficacy group. An operator with a sense of inability to face occupational problems spent significantly more hours at work than his colleagues. It should be recalled that this group consisted of respondents with the longest period of employment. This implies that the results obtained in this group indicate a particular type of burnout in the form of a sense of professional failure. It is all the more important as one study conducted in a group of medical staff working in intensive care units showed a negative relationship between the period of employment and emotional exhaustion, and no relationship at all between the period of employment and other dimensions of the construct [50]. It is known from scientific literature that prolonged working time poses a risk of insufficient time for regeneration of an employee’s psychophysical strength, which increases the risk of emotional exhaustion [7,33]. The number of working hours is not, however, the only predictor of exhaustion, which is indicated by the results obtained in the high burnout risk group. Although the level of burnout was the highest in this group, its members spent the lowest number of hours at work. It might be accounted by the relatively shortest working experience among the members of this group, which prevented them from developing sufficient personal resources (e.g., ways of coping with stressors) and, simultaneously, from the lack of sufficient job resources to protect the group of employees with low seniority.

As far as personal resources, it should be noted at the outset that the transactional burnout model has been based on theories about job stress, and the notion of imbalances leading to strain [51]. A significant role in a burnout model formulated in this way is played by appraising the work situation in terms of matching job demands with personal resources. As for the way of assessing the situation as stressful, the lack of occupational burnout symptoms coincided with the rate of respondents’ maintaining their efficacy in coping with work stressors and life problems and their ability to control them, and the sense of predictability of events and signs of endurance in case of emotional overload. This was observed in the no risk of burnout with an increased sense of disappointment group. The comparison of the obtained data with the results of other occupational groups leads to the conclusion that the levels of perceived stress in the burnout risk group and in the without full-blown pattern of burnout with high inefficacy group were noticeably higher than the level obtained in the study carried out on a group of Polish air traffic controllers and maritime navigators [48]. These professions are commonly regarded as highly stressful [52], and, at the same time, requiring particular personal predispositions from candidates which would make them more resistant to stressors. As stated by Cohen et al. [11], perceived stress can be viewed as an outcome variable measuring the experienced level of stress as a function of objective stressful events, coping processes, and personality factors. Even though the assessment of perceived stress is not a measure of psychological symptomatology, it can be used for recognizing people at risk of specific clinical mental disorders, including occupational burnout syndrome [53].

Returning to the results of our research, the causes of high levels of perceived stress in the burnout risk group and in the group of without full-blown pattern of burnout with high inefficacy can be sought in interactions between job demands and personal resources. One explanation is that the employees who perceived a situation as highly stressful were able to subjectively notice a growing number of demands in their work environment [54]. What arose at that moment was a spiral of negative changes: high level of stress—chronic exhaustion—deterioration in interpersonal relations—an increase in the demands of working environment—a decline in efficacy and commitment [55], which may explain the results in the high-risk group. In the case of the group without full-blown pattern of burnout with high inefficacy, a high level of perceived stress coexisted with only one dimension of burnout: professional inefficacy, perhaps due to the longest years of service as ECD. No increase in work resources may decrease the level of employees’ commitment and amplify the stressful impact of job demands [55].

It was observed that self-efficacy might contribute to work commitment, especially when the work was performed under conditions of strong emotional overload and other high work demands [17]. Such a situation occurs, among others, in the work environment of ECDs. According to suggestions put forward by Bakker and Demerouti [7], work resources might be interdependent with personal resources, which may indicate a feedback effect between variables, so personal resources may be predictors of organizational resources. The conducted study confirmed the hypothesis about the importance of the self-efficacy level for differentiating groups according to the intensity of symptoms of occupational burnout. Respondents from the no risk of burnout with an increased sense of disappointment group reported a significantly higher level of self-efficacy compared with those from the burnout risk group and the without full-blown pattern of burnout with high inefficacy group. Comparing the aforementioned results with the differences concerning the employment period, it may be assumed that the respondents from the burnout risk group, who, at the same time, had the shortest period of employment, maintained a self-efficacy level from the time before they started working as an ECD, whereas the respondents from the without full-blown pattern of burnout with high inefficacy group and with the longest period of employment, reported a level of efficacy which had been shaped throughout their entire period of employment. Prati et al. [56] observed that self-efficacy is an important resource in rescue workers. People tend to regulate their level and distribution of effort according to the effects they believe their actions have on the effort. Employees’ beliefs about their own efficacy are essential for their own perception of the context in which they work, especially when having to cope with highly demanding and potentially stressful job requirements [57,58]. In such cases, employees with a positive outlook on their own effectiveness adapt better to stressors at work [59]. On the contrary, those workers who consider themselves ineffective, attribute failures to their competency deficit, thereby increasing the feeling of ineffectiveness [60]. Employees with a high level of self-efficacy tend to perceive stressors as less threatening and easier to cope with, which may prevent them from developing occupational burnout syndrome [61]. Konopaske, Ivancevich and Matteson [62] observed that employees with a high level of self-efficacy feel more confident in their skills and abilities and are more likely to perceive potential stressors as challenges or opportunities. In contrast, those with a lower level of the aforementioned variable feel less confident in their skills and abilities and are doubtful about the success of the tasks performed [16].

### 4.5. Profiles Which Are at Risk of Occupational Burnout

The conducted research allowed the identification of groups of ECDs with the highest risk of burnout. The first group was characterized by a shorter work experience, predominance of women, the shortest working week in terms of working hours, average level of perceived stress and self-efficacy, and the average number of people engaged in an active hobby, from among all the respondents. The findings of the study support an assumption concerning an increase in burnout risk in people with a relatively short time of employment in the profession [5,25,63,64,65,66]. The causes of burnout can be sought in the fact that employees are disappointed with the reality of their work and in falsification of erroneous professional expectations. Expectations related to one’s beliefs are often underlying motives behind the choice of a profession. They maintain the relationship between the employee and their workplace, which does not only come down to a utilitarian exchange. Conversely, when professional resources and job demands conflict with an employee’s values, the employee may look for compromise solutions, which exacerbates burnout [49].

In the study, we discovered the without full-blown pattern of the burnout in employees with the longest period of employment, which manifested itself only in the form of an extremely acute sense of occupational failure in employees with the relatively longest period of employment in this profession. The group was also characterized by a clear dominance of women over men (the highest in the three separate groups), the longest working week in terms of working hours, the highest level of perceived stress, the lowest level of self-efficacy and the lowest number of people engaged in an active hobby, among all the respondents. Although in each group we observed the dominance of people with higher education, the number of these people in the discussed group was average. The higher level of burnout in the group of responders with a lower level of education is inconsistent with the results of some studies, which confirms the complex relationships between the variables [66]. It is not possible to come to conclusions that there is a correlation between burnout and an ECD’s sex just because this professional group is dominated by women. Nevertheless, comparing the stressors which appear in call-takers and dispatchers’ work (emotional overload, time pressure, shift work giving rise to work–home conflict and hindering relaxation) with the differences within emotional intelligence between women and men, the ability to recognize emotions, which is more common in women [67], and within the methods of coping with emotions [68], some conclusions about aforementioned relationships can be drawn. They are even more likely, as they are confirmed by the results of studies conducted in other professional groups, that women tend to be more emotionally exhausted than men [30,66]. Some traditions and values in general society may promote the conditions that cause women to feel overworked [69]. Additionally, the group with no signs of burnout, but with an increased sense of disappointment and lack of enthusiasm, cannot be considered exemplary. Although there were people with the lowest level of perceived stress and the highest level of self-efficacy, a relatively high level of job disappointment compared with other dimensions of burnout was noticeable. Understanding the reasons for this would require further, longitudinal research.

## 5. Organizational Indications

The use of the person-oriented approach in the study of occupational burnout shows a more diverse picture of the phenomenon compared with the variable-focused approach, and highlights specific groups at risk through specific burnout symptoms. Identifying a group of responders with signs of severe burnout in all dimensions and the shortest work experience, moderate level of perceived stress and self-efficacy, requires the use of a job resource, such as mentoring, to develop their personal resources. Identifying a group of responders with only one high level of burnout, professional inefficacy, and at the same the largest number of working hours per week, in addition to the highest level of perceived stress and the lowest sense of self-efficacy, allows for a more precise indication of the goals of preventive actions in the areas of job demands and job and personal resources. Employees with a permanently high level of stress (a sense of helplessness) and low level of self-efficacy are likely to not only need help to replenish their professional resources (feedback on the effects of undertaken actions, social support in the form of discussing particular interventions, and actions supporting their decision-making autonomy), and changes in job demands (number of working hours per week), but also actions aimed at strengthening personal factors which are responsible for approaching a problem as a threat rather than a challenge. In both the case of employees with a short employment period (high risk of burnout group) and those with a long employment period (without full-blown pattern of burnout with high inefficacy group) there is a need to take actions in order to maintain or increase the sense of efficacy in dealing with difficult situations and obstacles. For a group with no signs of burnout with an increased sense of disappointment, using employees as mentors and promoting job-crafting methods could prevent disappointment from deepening even further. The next step in prevention is identifying each employee’s burnout profile, considering organizational and personal variables (resilience, empathy, self-esteem, optimism, and social support) [9,15].

Currently, occupational burnout is referred to as the “epidemic of the present times” due to its widespread occurrence and negative impact on the health of the individual, lower work efficiency and staff fluctuation [70]. This phenomenon is also associated with interpersonal problems of workers, a greater number of errors at work, absenteeism and, consequently, significant financial costs of the work performed, in addition to mental and physical costs of employees [71]. Study results show that jobs connected with emergency services are characterized by the highest compensation rates paid to employees who developed mental disorders due to exposure to psychological trauma and stressors at work. People working in this profession are more likely to suffer from overweight, obesity and loss of physical fitness compared with the general population [22]. The person-oriented approach offers a real opportunity to help these workers by customizing support within each profile.

## 6. Limitations

The use of self-reporting questionnaires entails a risk of a number of measurement errors, such as reporting bias, method variance error, obtaining data from only one source, and an error of reverse causality. We did not obtain the data from all PSAP in Poland, which means that the results may not be representative for the whole population of ECDs. In order to be able to show directional relationships, further randomized, controlled experiments on longitudinal effects should be planned. Only such research may allow for detecting the changes that take place in the relationships between job demands and professional resources, and occupational burnout, with its health consequences. It could be important to control the effect of additional job variables, such as salary levels, participation in additional training, and more. Future studies may lend the opportunity to explain the nature of the relationships between burnout, job demands and job resources within each profile.

## Figures and Tables

**Table 1:** Characteristics of the variables applied in the study. *n* = 553.

Parameter	M ± SD
Age	34.43 ± 8.11
Sex (women) %	56.4
Years of service	4.46 ± 2.64
Number of shifts per month	14.60 ± 6.83
Number of working hours per week	43.33 ± 6.37
PE	20.86 ± 3.91
RD	20.09 ± 4.24
PI	22.95 ± 3.22
DI	18.48 ± 3.45
PSS-10	15.92 ± 6.73
GSES	30.96 ± 4.90

Note: Variables were expressed as: M = mean ± SD (standard deviation), PE—psychophysical exhaustion, RD—relation deterioration, PI—professional inefficacy, DI—disappointment, GSES—generalized self-efficacy scale, PSS-10—perceived stress scale.

**Table 2:** Characteristics of the 3 clusters. *n* = 553.

Parameter		Cluster					
		A (*n* = 189)	B (*n* = 182)	C (*n* = 182)	Kruskal–Wallis H Test	Dunn–Bonferroni Test	*p*
PE	M ± SD	23.78 ± 2.98	19.88 ± 3.05	18.84 ± 3.77	178.79	A > B > C	*p* < 0.015
RD	M ± SD	23.36 ± 3.35	19.04 ± 3.21	17.76 ± 3.9	186.46	A > B > C	*p* < 0.015
PI	M ± SD	24.12 ±2.81	23.77 ± 2.87	20.94 ± 3.03	126.34	A > B > C	*p* < 0.015
DI	M ± SD	20.6. 3 ± 2.86	17.17 ± 2.77	17.58 ± 3.5	205.70	A > C > B	*p* < 0.015
LBQ^INDEX^	M ± SD	91.883 ± 5.80	79.857 ± 5.96	75.120 ± 9.49	340.901	A > B > C	*p* < 0.015

Note: Variables were expressed as: M = mean ± SD = standard deviation, PSS-10—perceived stress scale, PE—psychophysical exhaustion, RD—relation deterioration, PI—professional inefficacy, DI—disappointment. LBQ^INDEX^—link burnout inventory composite index of occupational burnout syndrome. Nonparametric statistics were used: Kruskal–Wallis H test, post-hoc Dunn–Bonferroni test for multiple analyses, *p* < 0.015.

**Table 3:** Comparison of cluster scores according to explanatory variables applied in the study. *n* = 553.

Parameter		Cluster					*p*
		A	B	C	Kruskal–Wallis H Test	Dunn–Bonferroni Test	
Years of service as ECD	M ± SD	3.19 ± 2.4	5.66 ± 2.48	4.2 ± 2.58	73.51	B > C > A	*p* < 0.015
Age	M ± SD	34.021 ± 8.75	34.937 ± 7.44	34.021 ± 8.75	3.095		n.s.
Sex %	Men	40.21	35.16	55.49	16.59	C > A > B	*p* < 0.015
	Women	59.79	64.84	44.51		B > A > C	
Number of shifts per month	M ± SD	14.27± 2.37	14.42 ± 1.88	14 ± 1.85	0.783		n.s.
Number of working hours per week	M ± SD	42.27± 6.52	45.08± 5.81	42.49± 6.43	36.37	B > C > A	*p* < 0.015
PSS-10	M ± SD	15.6 ± 5.86	21.42 ± 4.92	10.76 ± 4.61	237.63	B > A > C	*p* < 0.015
Education %	Vocational	0.53	0.00	0.00	5.99		*p* = 0.735^8^
	Secondary	31.75	23.63	21.98		A > B > C	
	Higher	67.20	75.27	77.47		C > B > A	
	No information	0.53	1.10	0.55			
Trained profession or only ECD profession	%	30	34.8	32,4			*p* = n.s.
GSES	M ± SD	31.35 ± 4.66	28.54 ± 5.34	32.98 ± 3.47	89.56	C > A > B	*p* < 0.015
Hobby, yes	%	93.6	84.92	95.1	18.28	C > A > B	*p* < 0.015

Note: Variables were expressed as: M = mean ± SD (standard deviation). Nonparametric statistics were used: Kruskal–Wallis H test, post-hoc Dunn–Bonferroni test for multiple analyses, *p* < 0.015. PSS-10—perceived stress scale, GSES—generalized self-efficacy.

## Data Availability

To obtain access to the data, please contact the corresponding author.
